# The Effective Role of Natural Product Berberine in Modulating Oxidative Stress and Inflammation Related Atherosclerosis: Novel Insights Into the Gut-Heart Axis Evidenced by Genetic Sequencing Analysis

**DOI:** 10.3389/fphar.2021.764994

**Published:** 2021-12-22

**Authors:** Richard Y. Cao, Ying Zhang, Zhen Feng, Siyu Liu, Yifan Liu, Hongchao Zheng, Jian Yang

**Affiliations:** ^1^ CMVD Collaborative Program, Shanghai Xuhui Central Hospital, Fudan University, Shanghai, China; ^2^ School of Sport Kinesiology, Shanghai University of Sport, Shanghai, China; ^3^ School of Medicine, Nantong University, Nantong, China

**Keywords:** berberine, oxidative stress, inflammation, atherosclerosis, gut microbiota, cardiovascular disease, natural product, Chinese medicine

## Abstract

The exacerbation of oxidative and inflammatory reactions has been involved in atherosclerotic cardiovascular diseases leading to morbidity and mortality worldwide. Discovering the underlying mechanisms and finding optimized curative approaches to control the global prevalence of cardiovascular diseases is needed. Growing evidence has demonstrated that gut microbiota is associated with the development of atherosclerosis, while berberine, a natural product exhibits antiatherogenic effects in clinical and pre-clinical studies, which implies a potential link between berberine and gut microbiota. In light of these novel discoveries, evidence of the role of berberine in modulating atherosclerosis with a specific focus on its interaction with gut microbiota is collected. This review synthesizes and summarizes antioxidant and anti-inflammatory effects of berberine on combating atherosclerosis experimentally and clinically, explores the interaction between berberine and intestinal microbiota comprehensively, and provides novel insights of berberine in managing atherosclerotic cardiovascular diseases via targeting the gut-heart axis mechanistically. The phenomenon of how berberine overcomes its weakness of poor bioavailability to conduct its antiatherogenic properties is also discussed and interpreted in this article. An in-depth understanding of this emerging area may contribute to identifying therapeutic potentials of medicinal plant and natural product derived pharmaceuticals for the prevention and treatment of atherosclerotic cardiovascular diseases in the future.

## Introduction

**FIGURE 1:**
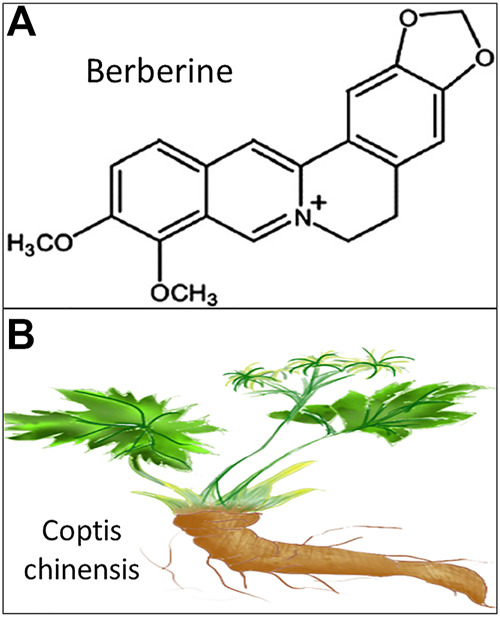
Schematic illustration of berberine. **(A)**. Chemical structure of berberine retrieved from National Center for Biotechnology Information https://pubchem.ncbi.nlm.nih.gov/compound/berberine; **(B)**. Herbal plant of Chinese medicine Coptis chinensis.

Medicinal plant-derived traditional Chinese medicines have recently received a lot more recognition likely as a result of the Nobel Prize winning discovery of natural product artemisinin. In recent years, emerging evidence has demonstrated that natural product berberine, an alkaloid from Chinese herb Coptis chinensis (Figure 1), exhibits a broad spectrum of biological activities in the prevention and treatment of cardiovascular diseases (CVD) (Cai et al., 2021). Nevertheless, the regulatory mechanisms of berberine involving CVD, especially how berberine influences CVD risk factor atherosclerosis, remain to be elucidated. Additionally, a growing number of novel findings have indicated that gut microbiota may be involved in facilitating the therapeutic effects of berberine against oxidative stress and inflammation (Cui et al., 2018; Wu et al., 2020). Therefore, an in-depth understanding of these frontiers may pave the way to develop more effective pharmaceutical approaches from botanical medicinal plants and bioactive natural products for the management of CVD patients with much less side effects.

Relevant information of berberine in modulating oxidative stress and inflammation related atherosclerosis was searched from the established academic databases such as PubMed, Web of Science, and ScienceDirect up to June 2021 with a focus on new findings in the past decade. Of course, several essential previous studies beyond that period and some most recent findings according to the reviewers’ suggestions were also included. Based on a special attention to the interaction between berberine and intestinal microbiota, search terms of berberine, oxidative stress, inflammation, atherosclerosis, gut microbiota, and combinations were used as key words.

Herein, the state-of-the-art discoveries in the past decade related to the role of berberine in atherosclerosis and the mechanisms of action are comprehensively reviewed in this article. The present review synthesizes and summarizes the antioxidant and anti-inflammatory effects of berberine on combating atherosclerosis, explores the dynamic interaction between berberine and intestinal microbiota, interprets the phenomenon of how berberine overcomes its weakness of poor bioavailability, and provides novel insights of berberine in managing atherosclerotic CVD via targeting the gut-heart axis.

## Effects of Berberine on Atherosclerosis

Atherosclerosis, a chronic inflammatory vascular disorder, is the primary cause of stroke, coronary heart disease, and acute myocardial infarction (AMI) leading to morbidity and mortality in developed and developing countries (Hansson, 2009). Cardiovascular risk factors such as hyperlipidemia, hyperglycemia, hypertension, obesity, physical inactivity, smoking, and aging promote vascular inflammation, endothelial dysfunction, and the progression of atherosclerotic CVD (Virani et al., 2021). Moreover, vascular oxidative stress plays a fundamental role in sub-endothelial retention of low-density lipoprotein (LDL) cholesterol, which undergoes oxidation within inflammatory macrophages eventually resulting in the accumulation of foam cells and the formation of atherosclerotic plaque (Skålén et al., 2002). Oxidative stress causes endothelial dysfunction, which also produces inflammation to accelerate plaque buildup. Activation of inflammatory cytokines secreted by macrophages may conceivably cause plaque rupture, thrombosis, ischemic symptoms, and even life-threatening consequences such as stroke and AMI. Currently, the possible measures for the prevention and treatment of atherosclerotic CVD are regular exercise, smoking cessation, and alternative medicines in addition to standard therapies to control oxidative stress and inflammation related atherosclerosis.

### Mechanisms of Berberine Involving Oxidative Stress and Inflammation

Mediators of oxidative stress and inflammation are closely related and can be important stimulators of atherosclerosis as above-mentioned. Berberine has been reported to modulate oxidative stress and inflammation through a variety of signaling pathways experimentally. Berberine was demonstrated to protect doxorubicin-induced cardiotoxicity against oxidative stress via suppressing Sirtuin one mediated p66^Shc^ signal, which was associated with reactive oxygen species (ROS) modulation, *in vivo* and *in vitro* (Wu et al., 2019). Moreover, berberine reversed homocysteine thiolactone induced ROS and promoted atherosclerotic plaque stability in Apoe^−/-^ mice with hyperhomocysteinemia through the mechanism of activating peroxisome proliferator-activated receptor gamma (PPAR*γ*), a transcriptional factor against oxidative stress and inflammation (Li et al., 2016; Idris-Khodja et al., 2017). Meanwhile, berberine decreased obesity-induced inflammation through suppression of endoplasmic reticulum stress and promotion of macrophage M2 polarization via down-regulating lncRNA Gomafu in obese mice and free fatty acids-treated adipocytes (Han et al., 2020). Berberine could increase energy expenditure and improve insulin sensitivity through uncoupling protein (UCP) 1/adenosine monophosphate-activated protein kinase (AMPK)/peroxisome proliferator-activated receptor gamma coactivator (PGC) 1*α* signaling pathways in atherosclerosis related obese mice (Zhang et al., 2014). Berberine also attenuated cardiac fibrosis and dysfunction through reducing cardiac insulin-like growth factor 1 receptor (IGF-1R) expression in diabetic rats, and inhibited inflammatory response via suppressing matrix metalloproteinase (MMP)-2/MMP-9, *α*-smooth muscle actin, and collagen type I expression in high glucose-cultured cardiac fibroblasts (Li et al., 2018). Novel evidence from a mouse model of anoxia-reoxygenation injury revealed that berberine could block inflammatory cytokine expression including interleukin (IL)-6, tumor necrosis factor (TNF)-α, IL-10 and IL-17A and cardiomyocyte apoptosis through down-regulating the p38 mitogen-activated protein kinase (MAPK)-induced nuclear factor-κB (NF-κB) signaling pathways (Zhao et al., 2019). Furthermore, berberine was discovered to reduce oxidative stress and vascular inflammation related atherogenesis via activating AMPK/UCP2 signaling pathways in a mouse model of atherosclerosis and cultured human umbilical vein endothelial cells (Wang et al., 2011). A most recent laboratory study proved that berberine could inhibit atherosclerosis by modulating autophagy, promoting cell proliferation and inhibiting cell apoptosis through regulating the phosphoinositide 3-kinase (PI3K)/Protein kinase B (AKT)/mammalian target of rapamycin (mTOR) molecular pathways in apoE knockout mice (Song and Chen, 2021).

**FIGURE 2:**
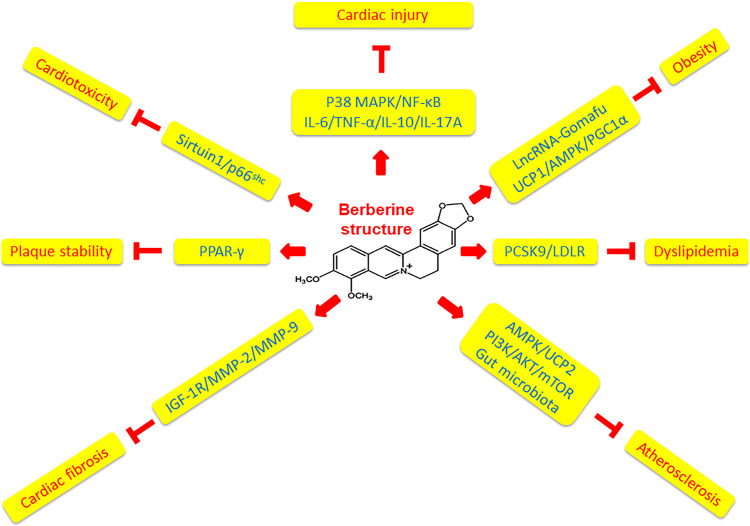
Mechanisms of berberine affecting atherosclerotic cardiovascular diseases. Berberine has pleiotropic effects on cardiovascular diseases *via* multiple mechanisms. Berberine protects cardiac injury by downregulating the expression of inflammatory cytokines including IL-6, TNF-α, IL-10 and IL-17A *via* p38 MAPK-mediated NF-κB signaling pathways. Berberine ameliorates obesity *via* downregulating lncRNA Gomafu and modulating UCP1/AMPK/PGC1*α* signaling pathways. Berberine inhibits dyslipidemia through regulating PCSK9/LDLR pathways. Berberine suppresses atherosclerosis *via* activating AMPK/UCP2 signaling pathways, regulating PI3K/AKT/mTOR molecular pathways, and modulating gut microbiota. Berberine reduces cardiac fibrosis *via* inhibiting IGF-1R/MMP-2/MMP-9 signaling pathways. Berberine activates PPAR*γ* to increase atherosclerotic plaque stability. Berberine ameliorates doxorubicin-induced cardiotoxicity through Sirtuin1/p66^shc^ pathways.

Collectively, in comparison to the more specific effect of artemisinin on malaria, berberine displays pleiotropic effects on cardiovascular and cardiometabolic diseases including dyslipidemia, hyperglycemia, hypertension, arrhythmia, and heart failure via multiple mechanisms (Cao et al., 2021). Feng and international collaborators provided a timely, in-depth and state-of-the-art review of basic, translational and clinical studies of berberine regarding novel molecular targets, such as AEBP1 (adipocyte enhancer-binding protein 1), HNF-4*α*(hepatocyte nuclear factor-4*α*), TRPV4 (transient receptor potential vanilloid 4), SIRT1 (silent information regulator 1), PTP1B (protein tyrosine phosphatase1B), etc., in a much wider aspect of cardiovascular and cardiometabolic diseases (Feng et al., 2019). Focusing on the role of berberine in modulating oxidative stress and inflammation related atherosclerosis in this review article, we have identified that berberine ameliorates doxorubicin-induced cardiotoxicity through Sirtuin1/p66^shc^ pathways, activates PPAR*γ* to increase atherosclerotic plaque stability, meliorates obesity via downregulating lncRNA Gomafu and modulating UCP1/AMPK/PGC1*α* signaling pathways, reduces cardiac fibrosis via inhibiting IGF-1R/MMP-2/MMP-9 pathways, protects cardiac injury by downregulating the expression of inflammatory cytokines including IL-6, TNF-α, IL-10 and IL-17A via p38 MAPK-mediated NF-κB molecular pathways, decreases atherogenesis via activating AMPK/UCP2 signaling pathways, and inhibits atherosclerosis through regulating PI3K/AKT/mTOR molecular pathways. The mechanistic effects of berberine on oxidative stress and inflammation are summarized in Table 1 and illustrated in Figure 2. Further investigations revealing the molecular mechanisms underlying the pleiotropic role of berberine by means of cell and animal models are required to enhance our knowledge in this field.

**TABLE 1:** Mechanisms of berberine involving oxidative stress and inflammation.

Animal and cell models	Regulatory targets	Signaling pathways	References
Cardiotoxicity rat and H9c2 cell	ROS, apoptosis, oxidative stress	Sirtuin-1/p66^shc^	Wu et al. (2019)
Atherosclerotic mouse	ROS, oxidative stress, inflammation	PPAR-*γ*	Li et al. (2016)
Obese mouse and adipocyte	ERS, macrophage M2 polarization, inflammation	LncRNA Gomafu	Han et al. (2020)
Obese mouse and adipocyte	Thermogenesis, insulin sensitivity	UCP1/AMPK/PGC1*α*	Zhang et al. (2014)
Diabetic rat and cardiac fibroblast	Cardiac fibrosis, *α*-SMA, collagen type I, inflammation	IGF-1R/MMP-2/MMP-9	Li et al (2018)
Myocardial injury mouse and cell	Apoptosis, inflammation	IL-6/TNF-α/IL-10/IL-17A P38 MAPK/NF-κB	Zhao et al. (2019)
Atherosclerotic mouse and HUVE cell	Oxidative stress, inflammation	AMPK/UCP2	Wang et al. (2011)
Atherosclerotic mouse	Autophagy, cell proliferation, apoptosis	PI3K/AKT/mTOR	Song and Chen, (2021)
Dyslipidemia mouse	Inflammatory cytokines, lipid profiles, 8-isoprostane	PCSK9/LDLR	Xiao et al. (2012)

AKT, protein kinase B; AMPK, adenosine monophosphate-activated protein kinase; ERS, endoplasmic reticulum stress; HUVE cell, human umbilical vein endothelial cell; IGF-1R, insulin-like growth factor-1 receptor; IL, interleukin; LDLR, low-density lipoprotein receptor; LncRNA, Long non-coding RNA; MAPK, mitogen-activated protein kinase; MMP, matrix metalloproteinase; mTOR, mammalian target Of rapamycin; NF-κB, nuclear factor-κB; PCSK9, proprotein convertase subtilisin/kexin 9; PGC1*α*, peroxisome proliferator-activated receptor gamma coactivator 1 *α*; PI3K, phosphoinositide 3-kinase; PPAR-*γ*, peroxisome proliferator-activated receptor-*γ*; ROS, reactive oxygen species; TNF-α, tumor necrosis factor-*α*; UCP, uncoupling protein.

Over the last few years, a cholesterol homeostasis regulatory molecule called “proprotein convertase subtilisin/kexin 9” (PCSK9) has emerged to be a hot therapeutic target for decreasing cardiovascular inflammation, reducing cholesterol levels, and controlling cardiovascular risks by the mechanism of preventing its interaction with LDL receptor (LDLR) (Bergeron et al., 2015; Murphy et al., 2019). Experimental studies manifested that berberine decreased PCSK9 mRNA and protein levels in a time- and dose-dependent manner in HepG2 cells and improved dyslipidemia with lipopolysaccharide-induced inflammation by way of modifying the PCSK9/LDLR signaling pathway in C57BL/6 mice (Cameron et al., 2008; Xiao et al., 2012). Therefore, the discovery of the star molecule PCSK9 has enriched our understanding regarding the mechanism of berberine besides oxidative stress and inflammation (Table 1 and Figure 2). Furthermore, the mechanism of action by which berberine inhibiting PCSK9 transcription was demonstrated to be associated with down-regulation of PCSK9 obligated *trans*-activator hepatocyte nuclear factor 1α expression by virtue of ubiquitin-induced proteasomal degradation pathway *in vivo* and *in vitro* (Dong et al., 2015). It was reported that berberine derivatives might be more potent than berberine for down-regulating PCSK9 protein levels and promoting LDL cholesterol clearance in a most recent *in vitro* study (Fan et al., 2021). Nevertheless, in opposition to the above results that berberine down-regulating PCSK9 expression, a research team showed that berberine increased serum PCSK9 concentration in a dyslipidemia animal model of high fat diet-fed rats (Jia et al., 2014). In view of these contradictory results, thorough exploration is tremendously required to open up the truth underneath the complex situation.

### Berberine in Clinical Practice

Problems of inflammation, glucose and lipid dysmetabolism are regarded as main risk factors for endothelial dysfunction and progression of atherosclerosis leading to the development of life-threatening cardio-cerebrovascular complications. More and more studies suggest that berberine exhibits beneficial effects on protecting endothelial function and improving glucose and lipid profiles not only experimentally but also clinically (Pirillo and Catapano, 2015). In a study with twelve healthy volunteers receiving berberine oral administration at a dose of 1.2 g/day for 1-month in comparison to eleven healthy controls without berberine administration, berberine intervention was found to improve endothelial function in human subjects probably by the mechanism of diminishing microparticle-induced oxidative stress *via* regulating ROS and nitric oxide production tested in human umbilical vein endothelial cells (Cheng et al., 2013). In a recent clinical study of 40 patients with metabolic syndrome, oral administration of berberine at 1.2 g daily for 30 days displayed regulatory effects on blood glucose, lipids, and inflammatory parameters, for instance, high-sensitivity C-reactive protein (hs-CRP), IL-6 and TNF-α without more significant adverse drug reactions than those in the control group (Cao and Su, 2019). Another randomized controlled clinical trial with 100 patients randomly enrolled in the study (52 in the intervention group and 48 in the control group) after percutaneous coronary intervention, in which the experimental group received berberine treatment with an oral dose of 0.9 g/day for 15 days in addition to the regular medication, berberine promoted myocardial protection for postoperative patients through down-regulating inflammatory mediators including CRP, TNF-α, and IL-6 in plasma (Yao et al., 2018). Berberine also exhibited the effectiveness on improving lipid profiles including total cholesterol, LDL cholesterol, triglycerides, and high-density lipoprotein cholesterol in a double-blinded and placebo-controlled trial with 141 low cardiovascular risk patients (71 in berberine intervention group and 70 in placebo control group) at an oral dose of 0.5 g twice a day for 3 months (Derosa et al., 2013). The results were strictly confirmed by a “washout” design, in which the lipid profiles worsened when berberine was interrupted for 2 months during the washout period and then the lipid profiles improved again after the reintroduction of berberine for an additional 3 months in the above study. Moreover, berberine treatment at an oral dose of 0.3 g, tid, for 30 days in addition to the standard therapy significantly improved lipid profiles such as LDL cholesterol, triglycerides, and inflammatory parameters including MMP-9, intercellular adhesion molecule-1, vascular cell adhesion molecule-1 in 61 patients with acute coronary syndrome following percutaneous coronary intervention (Meng et al., 2012). In addition, there were no serious adverse events reported in above clinical studies.

In recent years, lipid-lowering nutraceuticals have been endorsed by guidelines and position papers from the international lipid expert panels for managing CVD residual risk to achieve CVD preventive goals (Cicero et al., 2017; Penson and Banach, 2020). Correspondingly, berberine-containing nutraceuticals were widely used in clinical practice for lipid management and reported in several clinical studies recently. For example, in a double-blind placebo-controlled clinical study of 60 patients with low-moderate risk hypercholesterolemia, the administration of a nutraceutical containing red yeast rice (200 mg, equivalent to 3 mg monacolins), policosanols (10 mg) and berberine (500 mg) combined with a hypolipidemic diet was found to be safe, tolerable, and effective to improve total cholesterol and LDL cholesterol at each time point of 4-, 12-, and 24-week treatment (Gonnelli et al., 2014). In an 8-week open-label single-arm pilot study of 40 patients with mild to moderate hypercholesterolemia, oral administration of the nutraceutical combination of fermented red rice (monacolin K 10 mg), liposomal berberine (47.2 mg), and curcumin (50 mg) once daily ameliorated lipid profiles such as total cholesterol, LDL cholesterol, oxidized (ox)-LDL, and triglycerides as well as reduced inflammatory parameters including hs-CRP and TNF-α (Biagi et al., 2018). In a recent study of 53 hypercholesterolemia patients with low to intermediate cardiovascular risk, 2-month treatment with the nutraceutical combination of monacolin K + KA (25 mg + 25 mg), berberine (500 mg), and silymarin (105 mg) reduced plasma LDL cholesterol and the inflammatory maker PCSK9 levels with a comparable result achieved by the synthetic lipid-lowering drug atorvastatin (Formisano et al., 2020). Additionally, in a parallel *in vitro* study, the same nutraceutical significantly reduced PCSK9 expression, lowered cholesterol levels, and suppressed foam cell formation in human macrophages (Formisano et al., 2020).

### Safety, Toxicity and Bioavailability Issues of Berberine

For the sake of public health, drug safety is an important issue that deserves attention. In general, above mentioned clinical studies regarding berberine and berberine-containing nutraceuticals did not report any serious adverse events. Thus, berberine and berberine-containing nutraceuticals present beneficial activities on modulating glucose, lipid profiles such as total cholesterol, LDL cholesterol, and triglycerides, reducing oxidative stress and inflammation parameters including ox-LDL, hs-CRP, IL-6, TNF-α, MMP-9, intercellular adhesion molecule-1, and vascular cell adhesion molecule-1 with good safety and tolerability. These results are in consistent with a meta-analysis involving 27 randomized controlled trials in which berberine is demonstrated to be effective and safe in the treatment of type 2 diabetes, hyperlipidemia and hypertension (Lan et al., 2015).

In addition to the pharmacological efficacy, safety and toxicity data of berberine should also be reviewed carefully. Though uncommon, a case report showed electrocardiogram sinus bradycardia, first-degree atrioventricular block and competitive junctional rhythm in a sportsman taking berberine for hypercholesterolemia indicating that berberine might have side effect in hypervagotonic persons (Cannillo et al., 2013). Additionally, berberine might increase risk for kernicterus in jaundiced newborn infants because administration of berberine resulted in a significant decrease in bilirubin serum protein binding due to displacement of bilirubin from albumin by berberine in an experimental study (Chan, 1993). Moreover, concomitant administration of berberine and macrolides and/or statins could enhance cardiac toxicity caused by inhibition of CYP3A4 and human ether-a-go-go related gene (hERG) channel because inhibition of CYP3A4 might elevate plasma drug concentration increasing cardiotoxicity and inhibition of hERG the potassium channel regulator might affect cardiac repolarization leading to long QT syndrome (Zhi et al., 2015; Feng et al., 2018a). Therefore, future studies related to clinical applications of berberine should be paid careful attention to safety and toxicity issues.

Notably, berberine has been increasingly recognized with its poor bioavailability after oral administration despite the multi-pharmacological effects on hyperglycemia and dyslipidemia (Liu et al., 2016). This interesting phenomenon of the comprehensive pharmacological effectiveness along with the low oral bioavailability and the possible explanation will be discussed further in the next section.

## The Interaction Between Berberine and Intestinal Microbiota

### Dysbiosis and Atherosclerotic CVD

Colonizing in the gastrointestinal tract, the microbiota plays an important role in maintaining host homeostasis (Lin and Zhang, 2017). However, a sequencing study on human samples of oral, gut, and plaque microbiota from patients with atherosclerosis found some bacterial taxa abundant in the gut sample were also plentiful in the atherosclerotic plaque within the same individual establishing a possibility of bacterial translocation from the gut to the heart (Koren et al., 2011). Correspondingly, emerging discoveries evidenced by genetic sequencing analyses have connected gut dysbiosis (an unbalanced gut microbiome) and atherosclerotic CVD (Sanchez-Rodriguez et al., 2020; Astudillo and Mayrovitz, 2021). For example, a recent metagenome-wide association study using next-generation sequencing technologies on fecal samples of 218 patients with atherosclerotic CVD and 187 healthy controls revealed that patients with atherosclerotic CVD had an enlarged cluster of Enterobacteriaceae and *Streptococcus* spp., which provided an extensive resource for further studies on the character of gut microbiome in health and disease (Jie et al., 2017). The connection of gut dysbiosis and atherosclerotic CVD is also verified in animal studies. In a rat model of AMI, significant composition changes of gut microbiota occurred at day 7 after the ligation of left anterior descending coronary artery, in which the abundance of *Synergistetes*, *Spirochaetes*, Lachnospiraceae, Syntrophomonadaceae*,* and *Tissierella Soehngenia* was observed in AMI group in comparison to sham control group (Wu et al., 2017). Another animal study showed that proinflammatory gut microbiota transplanted from caspase1^−/−^ mice increased systemic inflammation and promoted atherosclerosis in LDLR^−/-^ mice (Brandsma et al., 2019). The above findings establish a strong link between dysbiosis and atherosclerotic CVD implying that gut microbiota can be a potential target for new pharmaceutical development.

In addition to the composition alterations, metabolites of unbalanced gut microbiota also contribute significantly to human diseases (Lin and Zhang, 2017). Contemporary clinical and pre-clinical studies demonstrated that dietary l-carnitine, a nutrient with trimethylamine (TMA) abundant in red meat, was converted *via* gut microbiota-dependent mechanism to trimethylamine N-oxide (TMAO), a derivative from the metabolite TMA, more in omnivores than that in vegans/vegetarians along with TMAO further accelerating atherosclerosis (Koeth et al., 2013; Koeth et al., 2019). A screening of 79 DNA sequenced human intestinal isolates further illustrated that nine bacterial strains were able to produce proatherogenic metabolite TMAO from diet choline confirming the connection between gut microbiota and TMAO (Romano et al., 2015). Moreover, gut microbial metabolite TMAO was found to be associated with thrombus formation in 117 rheumatic heart disease patients with atrial fibrillation (Gong et al., 2019). TMAO was also found to enhance platelet hyper-reactivity and thrombosis risk in a cohort of 4,007 stable subjects presenting to a cardiology clinic for cardiac evaluation (Zhu et al., 2016). In the parallel animal studies, the role of gut microbiota in producing TMAO was further confirmed in germ-free and microbial transplanted mice (Zhu et al., 2016).

Taken everything into consideration, the above mentioned new discoveries have expanded our knowledge of how dysbiosis is linked to atherosclerotic CVD. Therefore, targeting gut microbiota and microbiota-derived metabolites will be a brilliant strategy to reduce cardiovascular risks for the prevention and treatment of CVD in the future.

### Berberine Ameliorates Dysmetabolism and Atherosclerosis *via* Interacting With Gut Microbiota

It has been reported that probiotics can influence the lipid metabolism and effectively alleviate atherosclerosis by means of regulating gut microbiota to reduce TMAO (Liang et al., 2020). As a promising therapeutic candidate for CVD, performing in a somewhat similar way like probiotics but not like antibiotics, berberine has exhibited its pharmacological effects on modulating dysmetabolism and atherosclerosis experimentally and clinically though its poor bioavailability is also perceived (Liu et al., 2016 ; Cao and Su, 2019; Cai et al., 2021). The possible explanation is that berberine displays its antiatherogenic properties via the active interaction with gut microbiota. This novel concept has been proposed in recent years (Habtemariam, 2020; Yang et al., 2021) and supported by several experimental studies (Feng et al., 2015; Feng et al., 2018a). For example, in an animal investigation using rats and mice, Feng and colleagues demonstrated that gut microbiota could metabolize berberine into its intestine-absorbable form of dihydroberberine, and this metabolite of berberine had a 5-fold higher intestine-absorbable rate than that of berberine origin (Feng et al., 2015). Additionally, the restriction of gut microbiota through antibiotics diminished the conversion of berberine to its metabolite dihydroberberine indicating the importance of gut microbiota in transforming berberine into its intestine-absorbable form in above study. Three years later, the same research team further investigated pharmacokinetics of berberine after oral administration in beagle dogs and discovered that eleven active berberine metabolites digested by gut microbiota were detected in plasma confirming the key role of gut microbiota in regulating berberine during its intestinal absorption process (Feng et al., 2018a). Besides, these results also support the theory that metabolites of berberine contribute to the multifunctional pharmacological activities in atherosclerosis.

Meanwhile, berberine was discovered to reduce obesity, restore the gut barrier, and suppress oxidative and inflammatory mediators including nicotinamide-adenine dinucleotide phosphate oxidase, lipopolysaccharide, TNF-α, IL-1β, monocyte chemotactic protein-1, etc. through modulating gut microbiota to reduce bacterial lipopolysaccharide levels in high fat diet induced dysmetabolic rats (Xu et al., 2017). Berberine also significantly affected gut microbiota and was found to reduce inflammation and atherosclerosis in high fat diet fed ApoE^−/-^ mice, accelerate the abundance of intestinal *Akkermansia spp*, and restore gut barrier integrity (Zhu et al., 2018). A similar animal study showed that berberine decreased atherosclerosis development and inflammatory cytokine expression on top of altered gut microbiota compositions and reduced TMAO levels (Shi et al., 2018). Moreover, a most recent study revealed that berberine diminished TMA/TMAO production and attenuated atherosclerotic lesions accompanying with the alteration of gut microbiota compositions such as Lachnospiraceae, Bacteroidales, Eubacterium, and also the above results were confirmed by transplantation of the TMA-producing bacteria in experimental mice (Li et al., 2021). Another recent investigation using high fat diet fed ApoE^−/-^ mice displayed that berberine exhibited pharmacological effects on minimizing atherosclerotic lesions, improving lipid profiles, and decreasing proinflammatory cytokines; additionally, berberine induced a beneficial phenotype of gut microbiota such as *Roseburia, Blautia, Allobaculum, Alistipes, Turicibacter,* and *Bilophila*, which promoted production of anti-inflammatory metabolite short-chain fatty acids (SCFA) and reduced the creation of atherogenic metabolite TMAO in a dose-dependent manner (Wu et al., 2020). A separate experiment with both *in vitro* and *in vivo* data showed that berberine promoted gut microbiota abundance of *Enterobacter, Escherichia-Shigella*, Incertae sedis, Lachnospiraceae *FCS020 group, Akkermansia, Clostridium sensu stricto 1, Bacteroides* to produce butyrate, a SCFA from dietary fibers with beneficial cardiovascular effects, which further improved blood lipid and glucose levels (Wang et al., 2017). Pretreatment with antibiotics in the above animal experiment eliminated the effect of berberine on promoting butyrate production implying that bioactive metabolites were key functional mediators, which resulted from the crosstalk between berberine and gut microbiota (Wang et al., 2017). The above results demonstrate that the regulatory role of berberine in dysmetabolism and atherosclerosis is via interacting with gut microbiota (Table 2).

**TABLE 2:** Berberine ameliorates dysmetabolism and atherosclerosis via interacting with gut microbiota.

Animal and cell models	Regulatory targets	Gut microbiota and metabolite changes	References
Obese rat with endotoxemia	Gut barrier, endotoxemia, oxidative stress and inflammation	Bacterial LPS	Xu et al. (2017)
Atherosclerotic mouse	Atherosclerosis, inflammation and gut barrier	*Akkermansia spp*.	Zhu et al. (2018)
Atherosclerotic mouse	Atherosclerosis, inflammation	*Firmicutes, Verrucomicrobia*, TMAO	Shi et al. (2018)
Atherosclerotic mouse	Atherosclerosis	Lachnospiraceae, Bacteroidales, Eubacterium, TMA/TMAO	Li et al. (2021)
Atherosclerotic mouse	Atherosclerosis, lipid profiles, inflammation	*Roseburia, Blautia, Allobaculum, Alistipes, Turicibacter, Bilophila,* SCFA, TMAO	Wu et al. (2020)
Obese mouse and rat	Lipid and glucose	Butyrate-producing bacteria including *Enterobacter, Escherichia−Shigella*, Incertae sedis, Lachnospiraceae *FCS020 group, Akkermansia, Clistridium sensu stricto 1, Bacteroides*; SCFA	Wang et al. (2017)

LPS, lipopolysaccharide; SCFA, short chain fatty acids; TMAO, trimethylamine N-oxide.

Taken together, on the one hand, gut microbiota converts berberine into intestine-absorbable metabolites to perform their therapeutic tasks. On the other hand, berberine changes gut microbiota compositions and metabolites into the antiatherogenic phenotype, which is beneficial for cardiovascular health. Therefore, the dynamic interaction between berberine and gut microbiota is extremely important for the prevention and treatment of oxidative stress and inflammation related atherosclerosis.

## Insights of Berberine Into the Gut-Heart Axis

By virtue of the fact that numerous gut microbiota metabolites are biologically active in regulating host pathophysiological status, thus gut microbiota also functions like an important endocrine organ that produces bioactive metabolites including TMAO, SCFA, branched-chain amino acids, bile acids, tryptophan, and indole derivatives to conduct their pathophysiological activities **(** Agus et al., 2021 **)**. For example, it was reported that gut microbiota metabolite TMAO accelerated atherosclerosis in mice (Koeth et al., 2013) and enhanced thrombosis risk in humans (Zhu et al., 2016), which might cause a heart attack or stroke. On the other side, antiatherogenic metabolite SCFA exhibited suppressive effects on hypertensive cardiac damage and atherosclerosis in experimental mice (Bartolomaeus et al., 2019) and protective effects on the progression of coronary artery calcification in humans (Ghosh et al., 2021).

On the basis of the next-generation genetic sequencing analysis (Koren et al., 2011; Jie et al., 2017; Wu et al., 2017; Brandsma et al., 2019), it is strongly evidenced that the intestinal microbiota communicates with the distal organ heart by means of complex pathways through not only bacterial translocation but also circulating metabolites and hence impacts phenotypes relevant to cardiovascular health and disease as described above. The arcane connection between the gut and the heart probably explains how gut bacteria and metabolites influence cardiovascular pathophysiology. Herein, the emerging gut-heart axis appears to be a novel pharmacological target for therapeutic exploration.

**FIGURE 3:**
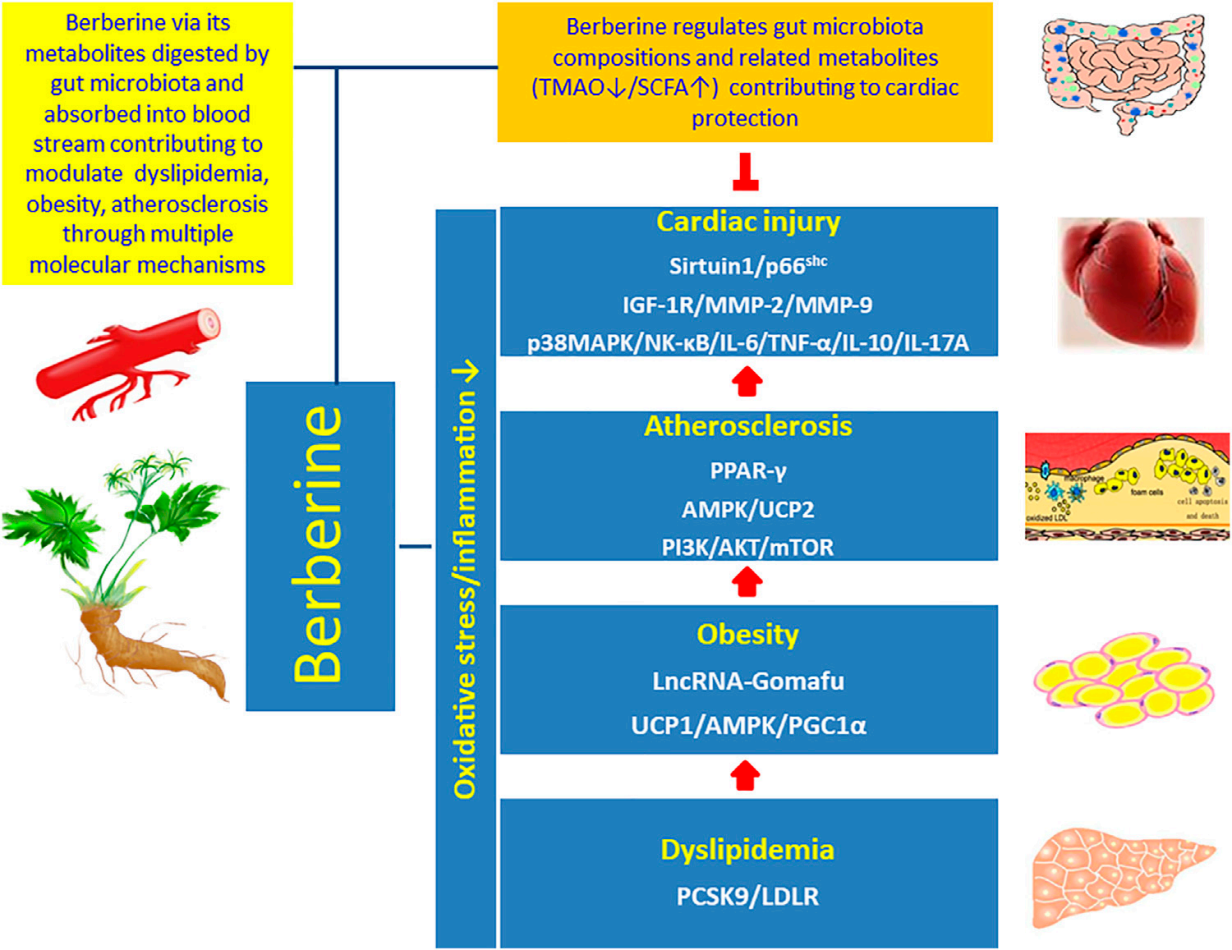
Interaction of berberine with the gut-heart axis. Berberine interacts with the gut-heart axis through two pathways: 1) berberine regulates gut microbiota compositions and related functional metabolites, for example inhibition of proatherogenic metabolite TMAO and promotion of antiatherogenic metabolite SCFA, which circulate to the distal organ heart and contribute to reduce coronary atherosclerosis; 2) berberine via its metabolites, digested by intestinal microbiota and absorbed into blood stream, contribute to modulate oxidative stress and inflammation related dyslipidemia, obesity, and atherosclerosis to inhibit cardiac injury via multiple molecular signaling pathways.

Berberine has been recognized as a pivotal therapeutic candidate for CVD because of its pleiotropic effects on modulating oxidative stress and inflammation related atherosclerosis (Cai et al., 2021). It has been proved that berberine interrupts atherosclerosis via manipulating gut microbiota compositions and functional metabolites (Wang et al., 2017; Agus et al., 2021), and meanwhile gut microbiota transforms berberine into intestinal absorbable form (Feng et al., 2015; Feng et al., 2018b). Accordingly, berberine and berberine metabolites conduct cardiac protective effects through two pathways directly and indirectly: 1) berberine regulates gut microbiota compositions and related functional metabolites, which circulate to the distal organ heart, to reduce coronary atherosclerosis; 2) berberine via its metabolites, digested by intestinal microbiota and absorbed into blood stream, contribute to protect the heart via multiple signaling mechanisms. Therefore, in both pathways berberine targets the gut-heart axis through the active interaction with gut microbiota to achieve the goal of cardiovascular protection (Figure 3).

## Conclusion and Future Perspectives

The beneficial impact of berberine on atherosclerotic CVD *via* modulating oxidative stress and inflammation is confirmed, and the interplay between berberine and gut microbiota at least partially unravels the phenomenon of how berberine overcomes its weakness of poor bioavailability after oral administration. Most importantly, like probiotics regulating gut microbiota rather than killing microbiota (Liang et al., 2020), berberine works differently from antibiotics by way of regulating gut microbes instead of eradicating them to avoid intestinal dysbiosis. Correspondingly, in consideration of the drug safety, no severe adverse reactions of berberine and berberine-containing nutraceuticals have been reported up to now (Meng et al., 2012; Biagi et al., 2018; Cao and Su, 2019; Chen et al., 2020) implying the potential value of berberine in clinical applications for the management of cardiovascular risk factors such as hyperlipidemia, hyperglycemia, hypertension, obesity, and atherosclerosis.

The interaction of berberine with gut microbiota reveals novel insights into the gut-heart axis, which opens up new avenues and concepts in utilizing medicinal plant and natural product derived pharmaceuticals to safeguard cardiovascular health. In view of the involvement of gut microbiota metabolites in the gut-heart axis, such circulating metabolites like TMAO and SCFA are acknowledged as potential biomarkers to reflect cardiovascular pathophysiological status. With the intention of having an in-depth understanding of this emerging area, further investigations by means of state-of-the-art technologies through incorporating pharmacodynamics, pharmacokinetics, microbiomics, and metabolomics are extraordinarily needed in order to develop novel therapeutic approaches to the prevention and treatment of atherosclerotic CVD in the future**.**
